# Auditory Information Accelerates the Visuomotor Reaction Speed of Elite Badminton Players in Multisensory Environments

**DOI:** 10.3389/fnhum.2021.779343

**Published:** 2021-11-25

**Authors:** Thorben Hülsdünker, David Riedel, Hannes Käsbauer, Diemo Ruhnow, Andreas Mierau

**Affiliations:** ^1^Department of Exercise and Sport Science, LUNEX International University of Health, Exercise and Sports, Differdange, Luxembourg; ^2^Luxembourg Health & Sport Sciences Research Institute A.s.b.l., Differdange, Luxembourg; ^3^Institute of Movement and Neurosciences, German Sport University Cologne, Cologne, Germany; ^4^German Badminton Association, Mülheim an der Ruhr, Germany

**Keywords:** EEG, athlete, sport performance, brain, training, vision, event-related potential

## Abstract

Although vision is the dominating sensory system in sports, many situations require multisensory integration. Faster processing of auditory information in the brain may facilitate time-critical abilities such as reaction speed however previous research was limited by generic auditory and visual stimuli that did not consider audio-visual characteristics in ecologically valid environments. This study investigated the reaction speed in response to sport-specific monosensory (visual and auditory) and multisensory (audio-visual) stimulation. Neurophysiological analyses identified the neural processes contributing to differences in reaction speed. Nineteen elite badminton players participated in this study. In a first recording phase, the sound profile and shuttle speed of smash and drop strokes were identified on a badminton court using high-speed video cameras and binaural recordings. The speed and sound characteristics were transferred into auditory and visual stimuli and presented in a lab-based experiment, where participants reacted in response to sport-specific monosensory or multisensory stimulation. Auditory signal presentation was delayed by 26 ms to account for realistic audio-visual signal interaction on the court. N1 and N2 event-related potentials as indicators of auditory and visual information perception/processing, respectively were identified using a 64-channel EEG. Despite the 26 ms delay, auditory reactions were significantly faster than visual reactions (236.6 ms vs. 287.7 ms, *p* < 0.001) but still slower when compared to multisensory stimulation (224.4 ms, *p* = 0.002). Across conditions response times to smashes were faster when compared to drops (233.2 ms, 265.9 ms, *p* < 0.001). Faster reactions were paralleled by a lower latency and higher amplitude of the auditory N1 and visual N2 potentials. The results emphasize the potential of auditory information to accelerate the reaction time in sport-specific multisensory situations. This highlights auditory processes as a promising target for training interventions in racquet sports.

## Introduction

High-level athletes participating in ball, team or racquet sports develop exceptional perceptual abilities to extract sensory information from the environment. While vision is the dominating sensory system athletes perform in multisensory environments where also auditory information play a performance determining role (Schaffert et al., 2019). In this context, auditory information have been shown to improve discrimination of shot power in soccer or volleyball (Sors et al., 2017), to predict attacks in fencing (Allerdissen et al., 2017) and anticipate movement behavior in basketball (Camponogara et al., 2017). Moreover, auditory information contributed to movement performance in continuous repetitive movements such as rowing (Schaffert et al., 2020) and was used in acoustic reafference training to improve motor learning of a hurdling task (Pizzera et al., 2017). Together, these findings emphasize the importance of auditory information in sports and the need for research investigating the interaction between visual and auditory information in realistic multisensory environments.

In racquet sports, visual information is accompanied by characteristic sounds at the ball racquet contact. When suppressing, masking, or manipulating these acoustic cues, perceptual-motor and sport performance are substantially affected. Performing sport-specific motor tasks in auditory deprived conditions resulted in lower precision performance in table tennis (Klein-Soetebier et al., 2020) and a higher rate of lost matches in tennis (Takeuchi, 1993). Similarly, masking natural auditory cues in tennis by grunting sounds systematically affected the anticipated ball trajectory dependent on grunt intensity (Müller et al., 2019) while a manipulation of audio-visual stimulus congruency and sound intensity delays the response time and anticipated length of volleyball serves and tennis strokes, respectively (Cañal-Bruland et al., 2018; Sors et al., 2018a).

The abovementioned studies focused on cognitively determined abilities, such as anticipation (Sors et al., 2018a; Müller et al., 2019) or complex visuomotor integration (Takeuchi, 1993; Klein-Soetebier et al., 2020). It remains unclear if auditory information also contributes to performance in more perceptually determined situations requiring rapid reactions. It is well established that athletes from visuomotor demanding disciplines such as volleyball (Zwierko et al., 2010), table tennis (Bhabhor et al., 2013), badminton (Hülsdünker et al., 2016, 2017), or soccer (Ando et al., 2001) exhibit faster visuomotor reactions when compared to non-athletes thus emphasizing its performance determining role. However, previous experiments were limited to monosensory visual stimulation while audio-visual interactions have not been investigated. Since especially in badminton different stroke types such as smash and drop evoke characteristic sounds, the question remains if these auditory cues can speed up the visuomotor reaction time.

From a behavioral and neurophysiological perspective, this may seem obvious. Auditory reaction times are well established to be around 20–40 ms faster when compared to visual reactions (Shelton and Kumar, 2010; Jain et al., 2015; Shaw et al., 2020). Accordingly, following the race model (Raab, 1962) also audio-visual reactions should be faster when compared to visual stimulation alone. However, based on the distance of about 9 m between badminton players, auditory when compared to visual stimulation is delayed by about 26 ms due to the comparatively low speed of sound waves (343.2 m⋅s^–1^) when compared to the speed of light (∼300,000,000 m⋅s^–1^). Further, the speed of audio-motor reaction depends on the stimulus intensity (Marshall and Brandt, 1980; Schlittenlacher et al., 2017), frequency (Santee and Kohfeld, 1977), rise time (Schlittenlacher and Ellermeier, 2015), and spectral complexity (Schlittenlacher et al., 2017). For instance, a decrease in sound intensity from 80 to 60 db (which is approximately the sound volume of a smash and drop in badminton, respectively) would result in a higher audio-motor reaction time of about 14 ms (Schlittenlacher et al., 2017). A similar pattern of results was also observed for sport-specific stimuli. Sors et al. (2018b) reported that manipulating the loudness of soccer kicks by 20 db was associated with a difference in reaction time of about 25 ms. Conversely, increasing the speed of a motion stimulation from 5 to 10 Hz reduces the visuomotor reaction time by about 30 ms (Hülsdünker et al., 2017).

If auditory information can accelerate the visuomotor reaction speed in sports substantially depends on the stimulus characteristics and cannot be answered by pure tones or generic visual stimulation that have been used in previous research. Instead, visual, and auditory stimulation must reflect realistic game situations including the visual speed, auditory profile and audio-visual delay to determine if faster auditory signal processing in the brain outweighs the delayed information perception. Further, it is still unclear if sport-specific audio-visual information induce a redundant signals effect (RSE) the faster reaction in multisensory when compared to monosensory conditions. If a RSE can be observed this would suggest sport-specific audio-visual interactions that go beyond a purely additive effect of visual and auditory information in a multisensory setting.

While monosensory and multisensory reactions were investigated on the behavioral level, the underlying neural process remained largely unconsidered. For the visual system previous research identified the N2 component of the motion onset visual evoked potential as a performance determining factor for visuomotor reaction speed (Hülsdünker et al., 2018, 2019). The N2 is observed around 170–250 ms following stimulus onset in the motion sensitive area MT and is suggested to reflect the perception and processing of visual motion information (Kuba et al., 2007; Hülsdünker et al., 2017). For auditory stimulation, variations in reaction time may be reflected by the N1 potential that is the largest and most reliable component of the auditory evoked potential and observed around 100 ms following stimulus onset (Legatt, 2015). Its latency parallels the decrease in reaction time with higher stimulation intensity (Jaskowski et al., 1994) and is accelerated in athletes when compared to non-athletes suggesting a performance-determining role in sports (Sharma et al., 2019). Differences in reaction time between stroke types and stimulation conditions may thus be reflected by variations in the latency of visual and auditory evoked potentials.

This study aims to evaluate if auditory information can speed up the visuomotor reaction time in ecologically valid multisensory environments. Nineteen high-level badminton players performed a reaction test in response to monosensory (visual, auditory) and multisensory (audio-visual) stimuli that were created based on sport-specific audio-visual characteristics of smash and drop strokes. In addition to reaction behavior, a 64-channel high-density electroencephalogram (EEG) was used to identify the neural activation pattern corresponding to mono- and multisensory stimulation.

It was hypothesized that even with a delay of 26 ms, auditory information should speed up the reaction speed for both, smash and drop conditions. Specifically, we expected that the faster processing speed in the brain should outweigh the initial transmission delay, which was based on the results of Shaw et al. (2020) reporting a difference of about 40 ms between purely visual and audio-visual reaction speed. Differences in reaction speed were expected being paralleled by variations in visual and auditory evoked potential latency.

## Materials and Methods

### Sample Size Calculation

Sample size was calculated for the outcome parameter reaction speed using the G^∗^Power (3.1.9.6) software package. In a recent study of Shaw et al. (2020), comparing auditory, visual and simultaneous audio-visual simple reaction time the effect with regards to audio-visual vs. visual reaction time was *d* = 1.8. Adding 26 ms resulted in an effect size of *d* = 1.0. Research on visual stimulation speed and sound pressure level that were also investigated in this study revealed effect sizes of *d* = 0.8 for stimulation speed (Hülsdünker et al., 2019) and η_p_^2^ = 0.58 for sound pressure level (Schlittenlacher et al., 2017). Since all studies revealed a high effect size and this study used a repeated measures ANOVA approach with 6 conditions [2 speed (fast slow) × 3 condition (audio, visual, and audio-visual)], a η_p_^2^ = 0.14 was used for sample size calculation (α-level < 0.05, test power of 0.90). This conservative calculation revealed a minimum number of 10 participants for this study.

### Subjects and Ethics

Nineteen high-level badminton players [age: 21 (±5) years, height: 177 (±11) cm, weight: 68 (±12) kg] from two federal badminton training centers in Germany participated in this study. Athletes had an average training experience of 13 (±5) years, play at the highest level in their age group and participate in national and international tournaments. All athletes were free of injury and experienced no pain, discomfort or limitation during their daily routines and exercise. Participants were provided the experimental protocol, and their written consent was obtained. In case of participants under the legal age, the consent form was signed by a parent or guardian. The study was approved by the local research ethics committee of the university in accordance with the declaration of Helsinki.

### Experimental Protocol

The experiment included a recording phase on a badminton court and a reaction test in the lab that were performed on different test days. The recording phase was conducted first to determine the ball speed and auditory profile of smash and drop strokes. This information was then transferred into visual and auditory stimuli used in the lab-experiment that assessed the effect of auditory information on the visuomotor reaction speed.

#### Field-Based Recording Phase

**FIGURE 1:**
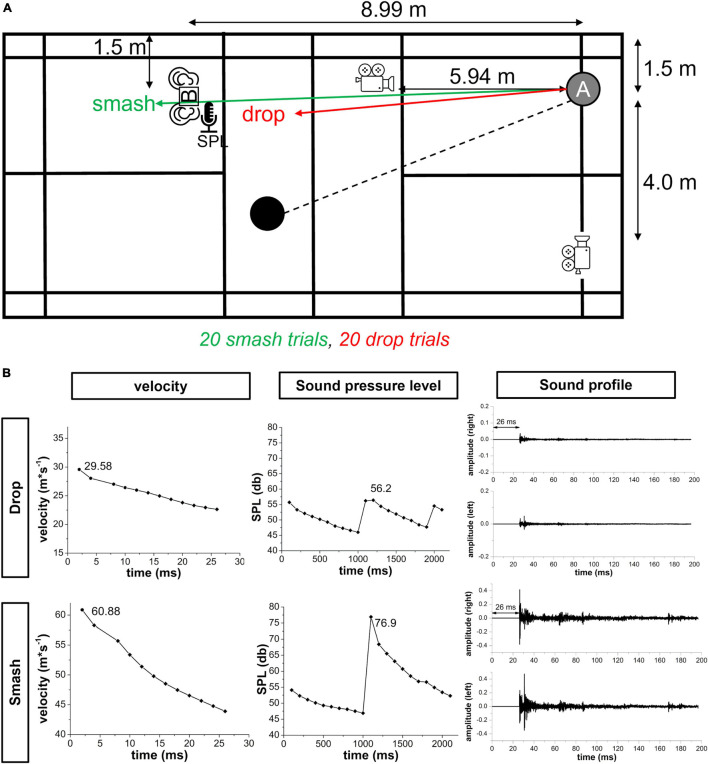
**(A)** Overview of the setup for the recording phase on the badminton court. Athletes played 20 smash and 20 drop strokes in a randomized order. High-speed cameras recorded the ball trajectory in the frontal and sagittal plane. The sound pressure level and binaural sound profile were measured using a sound level meter [SPL] and binaural microphones [B]. **(B)** Representative velocity sound pressure level and sound profile data of one participant for a smash and drop stroke that formed the basis for creating the visual and auditory stimuli in the reaction experiment. Note: the sound profile already includes the 26 ms silent period that account for the delay in auditory information transmission. Further, the scaling for smash and drop velocity is different.

The recording phase on the badminton court was designed to determine the parameters for the visual, auditory and audio-visual reaction tasks in the lab test. The setup for the recording phase is illustrated in Figure 1. Athletes played 20 smash and drop strokes in a randomized order. Players were informed about the stroke type prior to each trial. Balls were played by a second player at the other side of the court aiming for the long service line for doubles. Athletes were instructed to play longline without spin for both the smash and drop. For smash strokes, athletes should achieve the highest possible velocity while drops should be played close to the net. Movement behavior should reflect a real game situation. Prior to the test, each athlete performed at least 3 smashes and drops to become familiar with the task.

Smash and drop strokes were recorded using two Basler ace 800–510 uc (Basler AG, Ahrensburg, Germany) high-speed cameras with a spatial resolution of 800 × 600 pixels at a sampling rate of 500 Hz. Cameras were placed at an angle of 90° and captured the frontal and sagittal plane at a distance of 5.94 and 4.5 m and a height of 92 and 240 cm, respectively. A volume of 2 × 2 × 2 m was calibrated.

To determine the binaural sound profile smash and drop strokes, a 3Dio Free Space Pro II binaural microphone (3Dio, Vancouver, WA, United States) was used. The binaural microphone was mounted on a tripod and placed at a distance of 8.99 m to the attacking player simulating a realistic smash defense situation. The height of the binaural microphone was individually adjusted to the attacking player’s ears’ height in a defensive position to ensure a realistic sound profile. Sound data from both ears were amplified in a Focusrite Scarlett 2i2 amplifier (Focusrite Audio Engineering Limited Windsor House, Turnpike, United Kingdom) and recorded by the Audacity software package (Version 2.3.3.) at a sampling rate of 44.1 kHz. Prior to each participant both binaural microphones were calibrated using a PeakTech 8010 sound pressure level calibrator (PeakTech GmbH, Ahrensburg, Germany). The amplification of the Focusrite amplifier was adjusted based on the sound level during the familiarization trials.

Since digital signal amplitude does not provide an absolute measure of the sound pressure level, a PeakTech PCE-322A sound level meter was used to determine the absolute sound pressure level of smash and block strokes. The device was mounted on a tripod at the same height as the binaural microphones. The microphone was further inserted into a realistic rubber ear to maximize the validity of the measurement. Absolute sound pressure level was recorded using the Sound Level Meter (V3.4.1) software (PeakTech) with a sampling rate of 10 Hz.

To select a representative smash and drop trial for the visual, auditory and audio-visual stimulation in the lab-test, the smash and drop with the median sound pressure level was selected. Using the skillSpector 1.3 software package, the maximum velocity in x-direction of the ball within the first 10 ms following ball-racquet contact was calculated that formed the basis for the visual stimulus definition. The corresponding sound profile was transferred into a WAV file (44.1 kHz) that was used for auditory stimulation. Sound onset was defined as the first increase in signal amplitude during the ball-racquet contact and was determined visually. A silent period of 26 ms was added in the beginning reflecting the transmission time of a sound wave (343.2 m⋅s^–1^) to travel 8.99 m (distance between attacking player and binaural microphone).

Analysis of the field recording data resulted in an average velocity at ball-racquet contact of 27 (±5) m⋅s^–1^ and 80 (±14) m⋅s^–1^ for drop and smash strokes, respectively. The average sound pressure level for drop and smash was 61 (±2) db and 81 (±3) db. This information was used for the visual, auditory and audio-visual stimulation in the lab-test. The duration for the recording phase was about 20 min.

#### Lab Test (Reaction Task)

**FIGURE 2:**
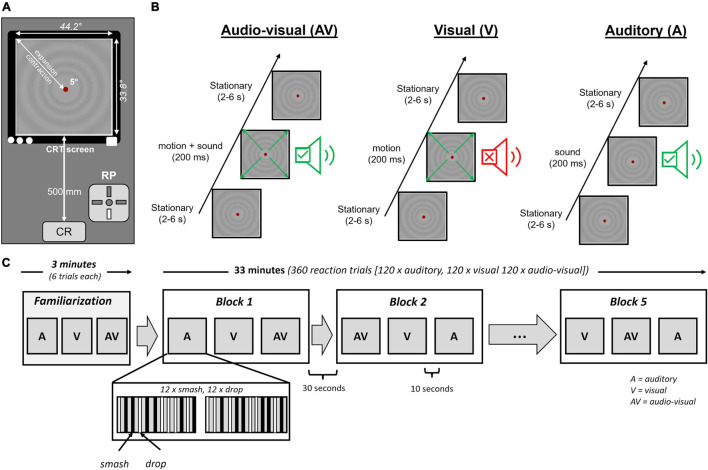
**(A)** Experimental setup for the reaction test in front of the computer screen. Auditory stimuli were presented using insert earphones. **(B)** Organization of stimulation for the audio-visual (AV), visual (V), and auditory (A) condition. Stationary periods randomly varied between 2 and 6 s to avoid temporal anticipation of stimulus onset. **(C)** Overview of the protocol for the auditory (A), visual (V), and audio-visual (AV) stimuli.

The lab test evaluated the reaction speed and neural activation profile in response to sport-specific visual, auditory, and audio-visual conditions. The setup for the lab-test is illustrated in Figure 2. To ensure normal vision and hearing, all participants completed a visual acuity (Landolt test) and hearing test at frequencies of 2k, 4k, 6k, and 8k Hz using a hearing test app (“Hearing Test”) together with Sennheiser HD 400S (Sennheiser GmbH, Wedemark, Germany) over-ear headphones. Participants were seated on a height-adjustable chair and placed their chin on a height-adjustable chinrest to ensure eyes were level with the screen center. The distance to the screen was 500 mm. Participants were instructed to focus on a red fixation dot, which was displayed during all conditions to avoid different environmental conditions and response strategies (e.g., closing eyes in the auditory condition). Therefore, even in the auditory condition the visual motion stimulus was displayed on the screen however remained stationary throughout the auditory reaction task. Eyes open/closed condition was continuously monitored based on the EEG data.

Participants performed visual, auditory and audio-visual reaction tasks in response to smash and drop strokes. Overall, 360 reaction trials were conducted [60 per condition (30 drop, 30 smash)], subdivided into 5 blocks of 3 conditions. Per block, the order of auditory, visual and audio-visual conditions was randomized. Each condition contained 12 smash and 12 drop strokes of one condition. The trials were organized in two sequences of 12 trials were the number of smash and drops was counterbalanced while presentation order was randomized. The pause between the two sequences within a block was 10 s. The pause between two blocks was 30 s. After the second and fourth block a pause of 1 min was included. Figure 2C illustrates the reaction protocol. Stimulus conditions (auditory, visual, and audio-visual) were not mixed within a single block to avoid task-mixing and task-switching effects (Shaw et al., 2020). The interstimulus interval randomly varied between 2 and 6 s (average = 4 s). Participants were instructed to press a button of a Cedrus RB-830 response pad (Cedrus, San Pedro, CA, United States) with the index finger of their dominant hand as fast as possible whenever they heard a sound or saw the circles move on the screen. The response pad signal was connected to the EEG amplifier and sampled at 1000 Hz. Before each block, participants were informed about the upcoming stimulus condition. Prior to the experiment, participants performed six trials of each condition to become familiar with the task. The duration of the stimulation protocol was about 33 min. The overall time for the lab test was about 90 min.

#### Visual Stimulation

Visual stimulation was induced by a radial motion onset stimulus that has previously been used in a series of experiments to identify the N2 component of the motion onset visual potential (Kuba et al., 2007; Hülsdünker et al., 2017, 2019, 2020). We decided to use this stimulus as it provides a high signal-to-noise ratio and allows a valid estimation of the N2 potential.

The velocity profile for the radial motion stimulus was calculated based on the aerodynamic properties of a badminton shuttle. Assuming an air density of 101.325 kPa (temperature of 20°C), a drag constant of 9.4 × 10^–4^ (Shibata et al., 2010) and a shuttlecock mass and skirt diameter of 54 g and 66 mm, respectively (Nakagawa et al., 2012) the distance between shuttlecock and player was calculated for every millisecond. Since the distance between object (shuttlecock) and players defines the visual angle on the retina the change in visual angle over time determines the expansion velocity of the motion onset stimulus. This expansion velocity was expressed in Hz and applied to the full field stimulation of the motion stimulus to keep the temporal frequency constant across the whole visual field (Kremlacek et al., 2004). To avoid adaptation effects visual motion stimuli were either contracting or expanding during the stimulation protocol. Importantly, the velocity of the visual stimulation was determined for each participant individually based on the velocity measured in the recording phase on the field. For each participant two stimulation profiles were determined, one for the smash and one for the drop condition to ensure an individual and realistic visual stimulation speed.

The visual stimuli were programed using the CRS toolbox (Cambridge Research Systems, Rochester, United Kingdom) implemented in Matlab (The Mathworks, Natick, MA, United States). A ViSaGe visual stimulus generator (Cambridge Research systems) presented the visual motion stimulus on a 22-inch HP1230 Color CRT monitor (Hewlett Packard Enterprise, San Jose, CA, United States) with a refresh rate of 120 Hz. The motion stimulus had a mean luminance of 17 cd⋅m^–2^. Stimulus luminance was sinusoidally modulated and had a maximum Michelson contrast of 10%. The visual stimulus subtended a visual field of 44.2° × 33.8°. A gray circle of 5° diameter was located at the screen center together with a red fixation point in the middle (both 17 cd⋅m^–2^). Prior to each participant, the screen luminance was calibrated using a ColorCalII. Frame synchronous stimulation was ensured by the ViSaGe system.

#### Auditory Stimulation

Auditory stimulation was delivered by the AudioFile system (Cambridge Research Systems). Simultaneously to the visual stimulation, an electrical impulse was sent by the ViSaGe to the AudioFile system that played the sound file derived from the field recording phase. Dependent on the condition (smash or drop), the corresponding sound file was selected. Like the visual stimulus, the auditory sound files had a length of 200 ms (including the 26 ms silent period). Therefore, the sound onset in the audio-visual condition started 26 ms later when compared to the visual motion onset which corresponds to lag of auditory when compared to visual information transfer. Etymotic ER-1 insert earphones with foam ear buds (Etymotic Research, Elk Grove Village, IL, United States) were used for auditory stimulation. Headphones were connected to a JDS Objective 2 pre-amplifier (JDS Lab, Collinsville, IL, United States). Prior to each participant, the amplification was adjusted that the output volume of the smash and drop strokes corresponded to the original sound pressure level determined in the recording phase on the badminton court. For calibrating the sound volume, the PeakTech PCE-322A sound level meter was used.

### EEG Acquisition and Analysis

EEG data was recorded using a 64-channel actiChamp amplifier (Brain Products GmbH, Gilching, Germany). Sixty-three active electrodes were equally distributed over both hemispheres according to the 10:10 system (Jurcak et al., 2007). One electrode was used to record electrooculographic signals. The ground and reference electrodes were placed on AFz and FCz, respectively. Electrode impedances were kept below 15 kΩ and data were recorded with an online low-pass filter of 280 Hz. Sampling rate was 1000 Hz. EEG data acquisition was synchronized by the electrical trigger pulse of the ViSaGe.

EEG data was analyzed in the Brain Vision Analyzer 2 software (Brain Products GmbH, Gilching Germany) and the EEGlab toolbox (Delorme and Makeig, 2004) implemented in Matlab. In a first step, EEG data was band-pass filtered between 0.3 and 35 Hz (zero phase-shift, IIR butterworth filter, notch filter at 50 Hz) and segmented into epochs of 1500 ms (−500 to 1000 ms relative to stimulation onset). Using an ocular correction ICA algorithm, blinks that occurred in an interval between −500 and 100 ms relative to stimulus onset were excluded. Moreover, a semiautomatic artifact rejection in an interval between −500 and 500 ms excluded segments with voltage steps >50 μV or a positive/negative amplitude exceeding ±150 μV. In addition, all segments were visually checked. On average, 9 (±4) segments were removed from the data. In EEGlab, an extended runica algorithm was used for ICA decomposition and the SASICA toolbox (Chaumon et al., 2015) identified artifactual components. Components were further visually checked based on time course, mapping and frequency spectrum and excluded if considered artifactual. On average 9 (±1) ICs were excluded prior to ICA back transform. Finally, a current source density transformation (number of splines = 4, maximal degrees legendre = 10, Lambda = 1e^–5^) was applied to the data.

To investigate event-related visual activity, this study focused on the N2 motion-onset visual evoked potential. Based on previous research, electrode positions corresponding to area MT (P7, P5, PO7, P6, P8, and PO8) were averaged and the N2 potential was identified as the maximum negative peak between 100 and 300 ms (Hülsdünker et al., 2019, 2020). For binaural auditory stimulation, the N1 component of the auditory evoked potential was identified by averaging electrode positions C3 and C4 that have previously been shown to show the sink maxima in auditory stimulation (Tenke and Kayser, 2012). N1 was defined as the maximum negative peak between 91 and 151 ms thus accounting for the 26 ms delay in auditory signal presentation (typical time window for N1 identification: 75–125 ms (Thaerig et al., 2008)). All identified potentials were visually checked.

### Statistics

Statistical analyses were performed in Statistica 7.1 (StatSoft, Tulsa, OK, United States). Normal distribution was confirmed (*p* > 0.05) for all variables using the Kolmogorov–Smirnov test.

To determine differences in reaction speed between auditory (A), visual (V), and audio-visual (AV) stimulation conditions, as well as between smash and drop strokes, a repeated measures ANOVA (RM-ANOVA) with the within-subject factors stimulation condition (auditory, visual, and audiovisual) and stroke type (drop, smash) was conducted.

Since visual and auditory evoked potentials were investigated in two conditions, amplitude and latency of the visual (N2) and auditory (N1) were compared in separate RM-ANOVAs with the within-subject factors stimulation condition (visual, audiovisual) and stroke type (drop, smash) for visual ERPs and stimulation condition (auditory, audiovisual) and stroke type (drop, smash) for the auditory ERPs. Bonferroni-corrected *post hoc* tests were applied to significant main and interaction effects.

To determine cortical processes contributing to the observed RSE, neural activity was compared between the multisensory condition (AV) and the summed activity of the auditory (A) and visual (V) condition (A + V) (Molholm et al., 2002). Multiple *t*-tests were calculated in a time interval between 50 and 200 ms following stimulus onset. Based on the reaction speed observed in the multisensory condition, this period was considered relevant for neural processes related to the sensory perception and initiation of the motor response. The false discovery rate procedure (Benjamini and Hochberg, 1995) was used to correct for multiple comparisons. Due to the better signal-to-noise ratio, this analysis was only conducted for the smash condition. Based on the cortical mapping of difference between AV and A + V, electrode position Cz as representative for pre- and supplementary as well as primary motor regions (Koessler et al., 2009) was considered in addition to electrodes determined for auditory (N1) and visual (N2) processes. Based on Cohen (1988), effect sizes were considered small (η_p_^2^ = 0.01), medium (η_p_^2^ = 0.06), or large (η_p_^2^ = 0.14). Significance levels were defined as follows: ^∗^ = *p* < 0.05; ^∗∗^ = *p* < 0.01; ^∗∗∗^ = *p* < 0.001.

## Results

### Reaction Time

**FIGURE 3:**
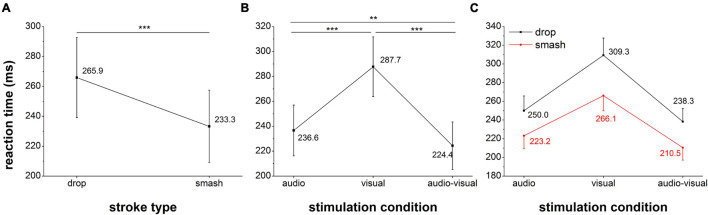
Reaction time analysis for the factor stroke type **(A)**, stimulation condition **(B)** and the interaction between both factors **(C)**. Error bars reflect 95% confidence intervals. **: *p* < 0.01, ***: *p* < 0.001.

The results for the reaction analysis are presented in Figure 3. The repeated measures ANOVA revealed significant main effects for stimulation condition (*F*_2,36_ = 211.7, *p* < 0.001,η_*p*_^2^ = 0.92) and stroke type (*F*_1,18_ = 166.5, *p* < 0.001,η_*p*_^2^ = 0.90) as well as a significant interaction (*F_2,36_ = 18.3, p* < 0.001, η_*p*_^2^ = 0.50). *Post hoc* test indicated the fastest reactions in the audio-visual condition (AV vs. V: *p* < 0.001, AV vs. A: *p* = 0.002) as well as faster auditory when compared to visual reaction speed (*p* < 0.001). The significant interaction effect between stroke type and stimulation condition (*F_2,36_ = 18.3, p* < 0.001, η_*p*_^2^ = 0.50) suggested are more pronounced acceleration in reaction speed from the drop to the smash condition in the visual when compared to the auditory and audio-visual condition.

### Auditory Evoked N1 Amplitude and Latency

**FIGURE 4:**
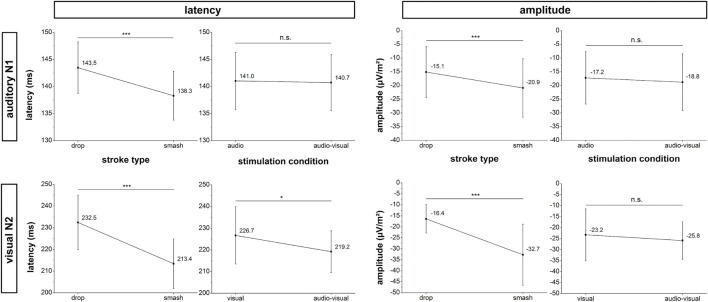
Amplitude and latency analyses for the auditory N1 (top row) and visual N2 (bottom row) potential. Error bars reflect 95% confidence intervals. *: *p* < 0.05, ***: *p* < 0.001, n.s.: not significant.

The temporal profiles and mappings of cortical activity for the visual and auditory event-related potentials of interest are presented in Figure 4.

Neurophysiological analyses focusing on the N1 latency of the auditory evoked potential yielded a significant main effect for stroke type (*F*_1,18_ = 17.1, *p* < 0.001, η_*p*_^2^ = 0.49) as characterized by a lower latency in the smash when compared to the drop condition. Main effects for stimulation condition (*F*_1,18_ = 0.2, *p* = 0.888, η_*p*_^2^ = 0.001) and the interaction between stimulation condition and stroke type (*F*_1,18_ = 0.1, *p* = 0.717, η_*p*_^2^ = 0.008) did not reach the significance level.

For the N1 amplitude, a main effect was only observed for the factor stroke type (*F*_1,18_ = 17.1, *p* < 0.001,η_*p*_^2^ = 0.48) indicating a significantly higher amplitude in the smash when compared to the drop condition. There was no main effect for stimulation condition (*F_1,18_* = 1.7, p = 0.207, η_*p*_^2^ = 0.09) or interaction between stimulation condition and stroke type (*F*_1,18_ = 0.001, p = 0.972, η_*p*_^2^ < 0.001).

### Visual Evoked N2 Amplitude and Latency

**FIGURE 5:**
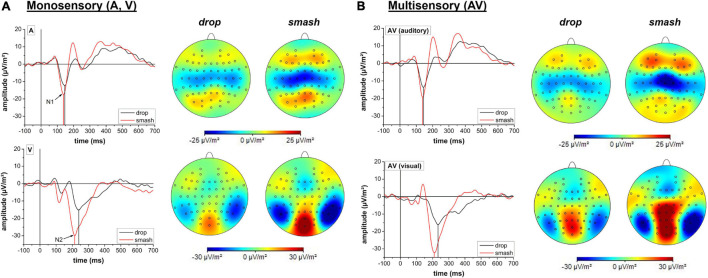
Temporal profile and cortical mapping reflecting auditory and visual activity in drop (black line) and smash (red line) strokes for monosensory **(A)** and multisensory **(B)** stimulation conditions. Cortical potentials of interest (N1, N2) are highlighted with arrows. Cortical mappings are based on a 10 ms peri-peak window centered at the N1 or N2 potential peak. Note different scaling for the mappings corresponding to the auditory N1 and visual N2.

Analyses of the visual evoked potential N2 latency revealed significant main effects for the factor stimulation condition (*F*_1,18_ = 5.9, p = 0.026, η_*p*_^2^ = 0.25) and stroke type (*F*_1,18_ = 25.2, p < 0.001, η_*p*_^2^ = 0.58) indicating a lower N2 latency in smash when compared to drop strokes as well as in the multisensory when compared to the visual-only condition. The interaction effect failed to reach the significance level (*F*_1,18_ = 0.5, p = 0.491, η_*p*_^2^ = 0.03). Data on N2 amplitude revealed a main effect for stroke type (*F*_1,18_ = 28.7, p < 0.001, η_*p*_^2^ = 0.61), indicating a higher N2 amplitude in the smash when compared to the drop condition. There were no effects of stimulation condition (*F*_1,18_ = 1.9, p = 0.179, η_*p*_^2^ = 0.10) or an interaction effect (*F*_1,18_ = 0.01, p = 0.915, η_*p*_^2^ < 0.001). Figure 5 illustrates the auditory N1 and visual N2 amplitudes and latencies across stroke types and stimulation conditions.

### Redundant Signals Effect

**FIGURE 6:**
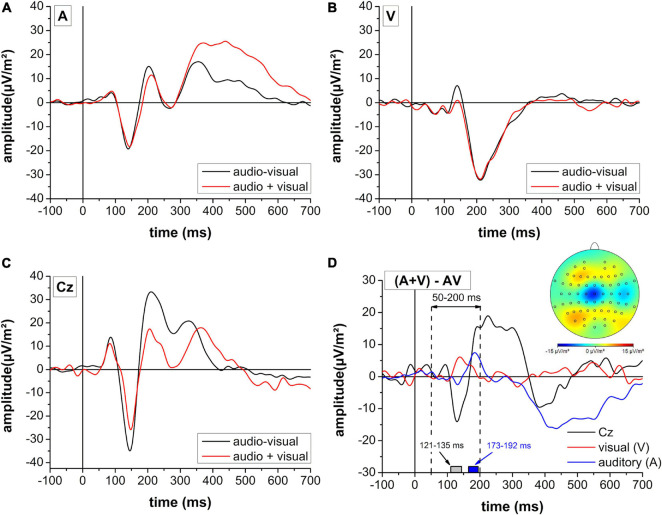
Contrast of summed monosensory (audio + visual: red line) and multisensory (audio-visual: black line) cortical activity in auditory **(A)** and visual **(B)** areas as well as electrode position Cz **(C)**. **(D)** Difference profile of the summed monosensory (audio + visual) and combined multisensory (audio-visual) activity for the three regions of interest. The cortical mapping reflects a 10 ms peri-peak window centered at the negative peak in electrode position Cz. Gray and blue boxes reflect the time periods of significant differences in electrode position Cz and the auditory region, respectively derived from the FDR-corrected multiple *t*-test analysis. Please note a different scaling for panel **(D)** when compared to panels **(A–C)**.

Results on the contrast between summed monosensory (A + V) and multisensory (audio-visual) stimulation are presented in Figure 6. In the time window between 50 and 200 ms following stimulus onset, a significantly stronger negative potential was observed in electrode position Cz between 121 and 135 ms following stimulus onset. For the auditory region, the multisensory stimulation revealed a higher activity between 173 and 192 ms while there was no effect for the visual area.

## Discussion

Considering the important role of auditory information in sport, this experiment investigated the reaction speed and neural activation in response to monosensory (auditory, visual) and multisensory (audio-visual) stimulation in elite badminton players. Sport-specific auditory and visual characteristics of smash and drop strokes were determined in a field-based recording phase and transferred into a reaction test allowing the investigation of behavioral and neural function under realistic stimulation conditions.

Superior reaction times in auditory when compared to visual stimulation suggest that the faster auditory signal processing in the brain outweighs the transmission delay of about 26 ms in badminton. Across stimulation conditions reactions were faster in response to smash strokes which was paralleled by faster neural signal processing and higher cortical activation as reflected by the N1 and N2 latency and amplitude. In accordance with previous research (Jaskowski et al., 1994; Hülsdünker et al., 2019) these findings indicate that the speed of neural sensory perception affects behavioral performance. The results further support a RSE as reflected by faster multisensory when compared to monosensory reaction. On the neural level this was primarily reflected by a stronger activation of motor areas probably reflecting a more efficient transfer of sensory information into a motor response.

### Monosensory and Multisensory Reaction Time

The primary objective of this study was to determine if sport-specific auditory information facilitate the reaction speed when compared to visual information in a sport-specific audio-visual setting. The results revealed that although the arrival of auditory information was delayed by 26 ms, the reaction was still about 50 ms faster when compared to the visual condition. This is in line with another sport-specific study by Sors et al. (2017) where response times for discriminating soccer penalty kicks and volleyball smashes were significantly faster with auditory or audio-visual when compared to only visual information. However, other experiments using generic auditory and visual stimuli (Shelton and Kumar, 2010; Jain et al., 2015; Jayaswal, 2016) typically quantify the differences between auditory and visual reaction speed in a range between 20 and 50 ms even without an auditory delay. The cumulative difference of about 76 ms (50 ms reaction time + 26 ms delay) in this study may thus be surprising but may be explained by the stimulus characteristics.

For the auditory reaction task, reaction times of 250 ms (drop) and 223 ms (smash) were well in accordance with the expectations for sound pressure levels of 60 db (drop) and 80 db (smash) (Schlittenlacher et al., 2017). Also in a sport-specific study on soccer penalty kicks, manipulating the sound pressure level by 20 db resulted in a reaction time difference of about 25 ms (Sors et al., 2018b). These findings further indicate that the difference in reaction speed between smash and drop strokes can be explained by the sound pressure level. Interestingly, previous research on simple visuomotor reaction time in badminton players revealed reaction times of 243 and 273 ms for a 5 Hz (slow) and 10 Hz (fast) visual motion stimulation (Hülsdünker et al., 2017) that was significantly faster when compared to the smash (265 ms) and drop (310 ms) strokes in this study. However due to the sport-specific velocity profile, the expansion/contraction speed of the stimulus in the beginning was lower when compared to previous stimulations at constant speed. Since stimulation velocity is directly related to reaction speed (Kawakami et al., 2002; Hülsdünker et al., 2019) the low initial stimulus speed delayed visual perception/processing [reflected by the comparatively high N2 latency (>200 ms)], and consequently the reaction time.

Reaction time analyses further supported a RSE as reflected by faster multisensory when compared to monosensory reactions. These findings are in line with previous studies reporting an RSE in audio-visual Go-NoGo (Minakata and Gondan, 2019) or decision making tests (Otto and Mamassian, 2012) but contrast the audio-visual simple reaction task by Shaw et al. (2020) where no difference between monosensory and multisensory reaction time was reported. Again, the stimulus characteristics may account for this discrepancy. While Shaw et al. (2020) used generic sound (1000 Hz) and visual (flashing red disk) stimuli, it was suggested that the RSE may be more apparent in complex multisensory environments as used by Minakata and Gondan (2019) who embedded the stimuli in random noise. The high complexity of sport-specific audio-visual stimulation with time-varying sound volume and frequency characteristics as well the non-linear velocity profile of visual stimulation may have contributed to the observed RSE. Another line of argument is the semantical congruence of the auditory and visual stimuli used in this experiment. As reported by Laurienti et al. (2004), semantically congruent stimulation improved feature discrimination performance. Importantly, this was only observed for cross-modal (audio-visual) but not unimodal (visual only) stimulation congruence. In contrast to generic stimuli, the auditory and visual stimulus characteristics in this experiment were interdependent, semantically congruent, and directly linked to smash and drop conditions in badminton. Further, since players were stimulated with the visual and auditory profiles of their own smash and drop strokes, the familiarity with the multisensory information may have facilitated the speed of perception. In fact, previous research suggested a greater perception accuracy with appropriate internal models (Schütz-Bosbach and Prinz, 2007; Kennel et al., 2014) that would be expected for high-level badminton athletes. Therefore, semantical congruence and familiarity with the stimulation characteristics may have facilitated the multisensory signal processing and accelerated the reaction time.

In sum, the behavioral results highlight the performance determining role of ecological auditory information for reaction speed in sport-specific multisensory environments. It supports previous behavioral research in sport science emphasizing the importance of ecological auditory information especially in racquet sports such as table tennis and tennis (Müller et al., 2019; Klein-Soetebier et al., 2020). The auditory system may thus be a relevant target for sport-specific training approaches which is in line with previous research on the positive effects of acoustic reafference training to improve motor performance (Pizzera et al., 2017). Advantages in multisensory when compared to monosensory reaction time further support the concept of RSE which may be related to the complexity and semantical congruence of the sport specific stimulation.

### Auditory and Visual-Evoked Potentials

Changes in reaction time were paralleled by modulations in the visual (N2) and auditory (N1) evoked potentials. In the smash condition, the N1 was characterized by a higher amplitude and lower latency. These findings are well in line with previous research likewise indicating a faster cortical activation (Lightfoot, 2016) and stronger neural response (Neuner et al., 2014) with increasing sound volume. This suggests a faster auditory signal perception and processing that in turn allows an earlier initiation of a motor response for the smash condition. A similar pattern of result was observed for N2 component of the motion onset visual-evoked potential indicating a lower N2 latency and higher N2 amplitude for the smash condition. Again, this supports previous research suggesting faster signal processing with increasing visual motion speed (Kawakami et al., 2002; Hülsdünker et al., 2019) that accelerates response initiation. Consequently, the results on auditory N1 and visual N2 potentials suggest that the higher sensory stimulus intensity in the smash condition (higher sound volume and movement speed), accelerates neural signal processing that improves the reaction time.

While there were substantial differences in neural activation based on stroke type, comparisons between the monosensory and multisensory conditions only revealed a significantly lower N2 latency in the audio-visual when compared to the visual condition. However, due to the latency of 219 ms that is even higher when compared to the multisensory reaction time in the smash condition, the N2 latency reduction in the multisensory condition cannot account for the RSE. Moreover, no effects were observed for the auditory evoked N1 amplitude and latency as well as the visual N2 amplitude which raises the question of the neural basis of the observed RSE in reaction time.

Molholm et al. (2002) analyzed the difference between the summed cortical activity of both monosensory conditions and the multisensory cortical response defined as the audio-visual interaction. It reflects activity pattern in the audio-visual condition that differs from a purely additive effect of auditory-only and visual-only stimulation and thus provides a potential explanation for the RSE. For this analysis, only the smash condition was selected since the higher sound volume evoked a higher amplitude and lower latency of the N1 potential. This has previously been shown indicative of a greater signal-to-noise ratio in auditory evoked potentials (Billings et al., 2009). Therefore, the smash condition was expected to generate more valid results for the RSE analysis. It was observed that the visual and auditory regions of interest revealed an almost identical activity profile for the summative (A + V) and multisensory (audio-visual) stimulation. In contrast, the cortical mapping of audio-visual interaction effects revealed a significantly higher negativity between 121 and 135 ms following stimulus onset with a peak at 128 ms on electrode position Cz.

This time frame corresponds to the centro-parietal audio-visual interaction reported by Molholm et al. (2002) about 120 ms following stimulation. We further follow the authors suggestion that the audio-visual interaction reflects a process different from auditory perception, although it overlaps with the auditory N1 peak. Specifically, while electrode position Cz reflected a stronger negativity around 130 ms, more nearby areas for the identification of the N1 potential (C3/C4) revealed a different temporal profile with higher positivity around 180 ms. Since electrode position Cz best represents pre- and supplementary as well as primary motor areas (Koessler et al., 2009), it may be speculated that motor processes contributed to the faster reaction in the audio-visual condition. In fact, an fMRI study by Calvert et al. (2001) has identified the pre- and supplementary motor cortex as one of the regions with audio-visual interaction effects. Further, a visual stimulation is accompanied by a widespread but unspecific cortical activation that not only includes visual but also motor areas (Ledberg et al., 2007). This visual-induced early motor activation may facilitate the transfer of auditory information into a motor command.

The combined pattern of neurophysiological findings supports the assumption that different reaction times between monosensory stimulation modalities (audio vs. visual) and stroke types (drop vs. smash) are reflected by the amplitude and especially the latency of the auditory and visual evoked potentials. In contrast, audio-visual interaction effects, were primarily observed around central motor areas. The transfer of sensory information into a motor response rather than purely sensory perception speed may contribute the RSE for reaction time.

### Limitations and Future Directions

In contrast to the auditory stimulation that matched the realistic sound profile in both volume and frequency spectrum, the radial motion onset visual stimulus was more generic and did not perfectly reflect real game characteristics. Specifically, to increase the signal-to-noise ratio for a valid N2 identification it was decided to use a full field stimulation and match the expansion/contraction speed to the horizontal velocity profile of the badminton shuttle. Consequently, the greater amount of motion information when compared to a badminton situation where only the shuttlecock moves in a stationary environment, the neural processing of visual information (N2 latency) and reaction speed may have been faster with the applied stimulation setting thus resulting in an overestimation of the sport-specific visual reaction speed. However, since the reaction in response to auditory information was still substantially faster this does not contradict but even emphasize the importance of auditory information in multisensory environments.

This study used a simple visuomotor reaction task. This setup has been selected to answer the question if and why the faster processing of auditory information outweighs the transmission delay in a realistic audio-visual environment. Therefore, cognitive processes were excluded in this experiment. This however contrasts many situations on a badminton court and other sports, that require rapid decision making based on audio-visual information.

To address these limitations, future research should expand the current findings to sport-specific choice-reaction and discrimination tasks investigating how incongruent audio-visual information affect reaction speed and discrimination performance. Moreover, incongruent audio-visual experiments with sound volumes matched to the visual stimulation speed may provide further information if the RSE depends on the pure existence of additional auditory information or its content. Based on the higher temporal acuity of auditory when compared to visual information (Merchant et al., 2015; Rammsayer et al., 2015) it may be argued that sounds provide a general advantage in multisensory environments. However, based on the heterogeneous results for generic stimuli (Minakata and Gondan, 2019; Shaw et al., 2020) and the proposed greater perception performance with existing internal models (Kennel et al., 2014) also a content-dependent RSE may be plausible. Moreover, behavioral experiments using more realistic visual stimuli (e.g., moving badminton shuttles instead of radial motion stimuli) may allow a better quantification of the auditory advantage in ecologically valid multisensory settings.

## Conclusion

Faster reactions in the auditory when compared to the visual condition even with a signal transmission delay of 26 ms support the relevance of acoustic information in high-speed racquet sport and emphasize the auditory system promising target for training interventions. Faster multisensory when compared to both monosensory reaction conditions add further support to the RSE. It suggests multisensory facilitation in a simulated sport-specific environment probably due to complex audio-visual stimulation or semantic congruence of auditory and visual information. Differences between stroke types and monosensory stimulation conditions were paralleled by modulations in auditory and visual evoked potential amplitude and latency adding further support to the performance determining of neural processes for reaction speed. However, the RSE on the behavioral level could neither be explained by audio-visual interactions in the auditory nor visual activity profile but may be related to a facilitation of sensory-motor transformation in motor areas.

## Data Availability Statement

The datasets presented in this article are not readily available because although personal data (e.g., name or address) of the participants are not included in the datasets, the risk of identifying athletes based on their age, gender, and sport is comparatively high since the groups of highly trained participants is small in badminton. This especially applies to the groups of young top-level athletes who are under legal age. Requests to access the datasets should be directed to TH, thorben.huelsduenker@lunexuniversity.net.

## Ethics Statement

The studies involving human participants were reviewed and approved by the Research Ethics Committee of the German Sport University Cologne. Written informed consent to participate in this study was provided by the participants’ legal guardian/next of kin.

## Author Contributions

TH, HK, and DRu organized the measurements at the badminton training centers. TH and DRi acquired the data and conducted the statistical analyses. TH wrote a first draft of the manuscript. DRi wrote sections of the manuscript. All authors contributed to the concept of this study, contributed to the revision of the manuscript draft, and read and approved the final version.

## Conflict of Interest

The authors declare that the research was conducted in the absence of any commercial or financial relationships that could be construed as a potential conflict of interest.

## Publisher’s Note

All claims expressed in this article are solely those of the authors and do not necessarily represent those of their affiliated organizations, or those of the publisher, the editors and the reviewers. Any product that may be evaluated in this article, or claim that may be made by its manufacturer, is not guaranteed or endorsed by the publisher.
